# Supplementary material for: Chemical Composition in Juvenile and Mature Wood of Branch and Main Trunk of Leucaena leucocephala (Lam.) de Wit
Source: Plants (Basel). 2023 Nov 26;12(23):3977. doi: 10.3390/plants12233977 (PMC10707876; doi:10.3390/plants12233977)
Supplement: Supplementary file 1 [file plants-12-03977-s001.zip › plants-2684870-SI.pdf]

Table S1. Band assignments of wood powder (WP) and Klason lignin (KL) from juvenile and mature wood of branch and main trunk of *Leucaena leucocephala*

No.	Wave number (cm^{-1}) Juvenile wood				Mature wood				Band origin, short comments references
	Branch		Main trunk		Branch		Main trunk		
	WP	KL	WP	KL	WP	KL	WP	KL	
1	3410	----	3413	----	3419	----	3412	----	O-H Stretching vibration [16]
2	2906	----	2922	----	2922	----	2922	----	C-H stretching vibration [16]
3	1738	1721	1738	1715	1739	1721	1739	1718	C=O Stretching vibration [16,17]
4	1639	1627	1628	1629	1634	1629	1639	1629	C=O stretching vibration in lignin [17]
5	1599	----	1599	----	1599	1542	1601	1541	Aromatic skelton vibration plus C=O stretch; S>G; G condensed > G etherified [16]
6	1510	1506	1510	1506	1509	1509	1510	1505	Aromatic skelton vibration, Benzene ring stretch in lignin(G>S) [17]
7	1461	1461	1461	1461	1462	1459	1461	1461	C-H Deformation vibration [16,17]
8	1425	1417	1424	1421	1425	1407	1424	1422	CH_2 scissor vibration and CH_3 bending vibration [16,17]
9	1375	-----	1374	-----	1375	-----	1375	-----	CH_3 bending [17]
10	1329	1323	1322	1322	1327	-----	1322	1322	S-ring + G-ring condensed in lignin [16]
11	1247	1285	1248	1284	1249	1282	1248	1285	CH3 COOR acetic ester (Upward direction) [16]
12	1161	1175	1159	1177	1160	1172	1160	1177	C-O-C antisymmetric bridge stretching vibration [16, 17]
13	1111	-----	1111	------	1113	-----	1111	-----	O-H association band [16]
14	1056	1069	1056	1069	1055	1058	1055	1069	C=O stretching [17]
15	1035	1010	1035	1010	1035	1026	1035	1010	aromatic C-H in plane deformation G>S plus C=O stretch [16]
16	897	885	886	885	898	882	885	885	Anomeric carbon group frequency [17]
